# A Factor Graph Description of Deep Temporal Active Inference

**DOI:** 10.3389/fncom.2017.00095

**Published:** 2017-10-18

**Authors:** Bert de Vries, Karl J. Friston

**Affiliations:** ^1^Department of Electrical Engineering, Eindhoven University of Technology, Eindhoven, Netherlands; ^2^GN Hearing Benelux BV, Eindhoven, Netherlands; ^3^Wellcome Trust Centre for Neuroimaging, Institute of Neurology, University College London, London, United Kingdom

**Keywords:** active inference, free-energy principle, factor graphs, belief propagation, message passing, multi-scale dynamical systems

## Abstract

Active inference is a corollary of the Free Energy Principle that prescribes how self-organizing biological agents interact with their environment. The study of active inference processes relies on the definition of a generative probabilistic model and a description of how a free energy functional is minimized by neuronal message passing under that model. This paper presents a tutorial introduction to specifying active inference processes by Forney-style factor graphs (FFG). The FFG framework provides both an insightful representation of the probabilistic model and a biologically plausible inference scheme that, in principle, can be automatically executed in a computer simulation. As an illustrative example, we present an FFG for a deep temporal active inference process. The graph clearly shows how policy selection by expected free energy minimization results from free energy minimization *per se*, in an appropriate generative policy model.

## 1. Introduction

Active inference is a corollary of the Free Energy Principle (FEP). The FEP argues from first principles that living systems retain their identities (i.e., stay alive) through the single mechanism of minimizing the variational free energy under a model of their environment (which is equivalent to maximizing the Bayesian evidence for that model) (Friston et al., 2006; Friston, 2012). Principally, implementation of active inference by a (biological or artificial) agent relies on only two procedures:

1. Model specification: The specification of a probabilistic generative model for the agent's sensory observations. Biologically, this starts with the genotype of the organism, as encoded by the DNA. For a scientist or engineer who wants to simulate an active inference process, this task involves the actual specification of the probabilistic model under study.
2. Free energy minimization: Once the model has been specified, the agent processes sensory observations exclusively through variational free energy minimization (VFEM). Free energy minimization is the agent's tool to approximately maximize the (Bayesian) evidence for its model of the environment, since explicit evidence maximization is computationally intractable. Crucially, the same VFEM process is used to infer all latent variables, including those that pertain to perceptive processes, action selection, attention mechanisms, memory creation and model pruning. In order to execute VFEM, the agent also needs access to a “proposal” distribution (usually a mean field version or factorization of the generative model) but in practice this distribution is relatively easy to specify once the generative model has been defined.

The second task, free energy minimization, is in principle an automatable process since the cost functional to be minimized is fully specified by the generative and proposal models (in conjunction with sensory observations). In biological systems, model specification (the first task) occurs through evolutionary refinement by natural selection, which is also a manifestation of free energy minimization at a large time scale (Harper, 2009; Campbell, 2016). As a result, active inference by the FEP is a fully automated (i.e., self-organized) process in biological systems.

For scientists who aim to simulate active inference processes, it would be of great help to have access to a “VFEM software toolbox” that automates the inference process for a wide range of probabilistic models. If such a toolbox were available, the work flow of the scientist would consist of proposing new candidate models and evaluating the performance of these models by calling the appropriate functions in the VFEM toolbox. In absence of such a VFEM toolbox, the scientist would likely be forced to derive the VFEM model-specific update equations by hand, which—for large models—quickly becomes an almost insurmountable obstacle. Thus, the speed and quality of synthetic active inference rests on the lubrication of the inference tasks that accompany the analysis of candidate models.

The impact of automated inference tools extends beyond fast simulations to support the study of biological active inference processes *per se*. Active inference by itself is a model for an automated scientific inquiry process, where all tasks (trial design, trial execution, performance assessment and adaptation) are executed as inference tasks on a probabilistic model. From an engineer's viewpoint, it is an enticing design strategy to develop active inference-based artificial agents that learn purposeful behavior (e.g., an audio or video processing task) from situated interactions with the environment (e.g., Van de Laar and De Vries, 2016).

The potentially large impact of automating probabilistic inference by a software toolbox is also recognized in the machine learning community. Under the header of “probabilistic programming,” various initiatives to develop toolboxes for automated inference are currently underway (Lunn et al., 2000; Minka et al., 2014; Salvatier et al., 2016; Tran et al., 2016; Carpenter et al., 2017). In particular recent work on black-box variational inference (BBVI) is interesting in the context of automating inference simulations (Ranganath et al., 2014; Taylor, 2016; Tran et al., 2016). When studying the brain however, we are not just interested in automating inference, but also in a biologically plausible realization of these inference processes. This feature is not a hard criterion in the current research lines on BBVI.

Rather than relying on automated-inference toolboxes, there have been attempts to develop a biologically viable process theory for active inference processes (Bastos et al., 2012; Friston et al., 2017a). These theories are accompanied by freely available software simulations in the “SPM toolbox,” (Friston, 2014). The SPM toolbox does support a wide range of demonstrations but does not support a scripting language for specifying novel candidate models with automated-inference support. Recent work has focused on graphical model descriptions of active inference processes (Friston et al., 2017c). The current paper provides a tutorial introduction to one variant of these graphical models.

In this paper we present Forney-style Factor Graphs (FFG) as a tool that supports both a visual representation of freely definable generative models and inference automation by biologically plausible message passing algorithms (Forney, 2001). Forney-style factor graphs are a type of graphical model that shares qualities with similar frameworks such as Bayesian networks and Markov random fields (Koller and Friedman, 2009). FFG graphs afford a visually insightful representation of the generative model, which is especially beneficial for complex models that underlie hierarchical active inference processes (preview Figure **7** for an example). In contrast to Bayesian networks and BBVI tools, FFGs also provide a precise view and description of a message passing-based inference process. As such, inference in an FFG furnishes a normative description of how biological neuronal inference processes might be executed (at a computational abstraction level).

FFGs were originally developed as a graphical framework for automating inference-based (de-)coding processes on graphs. About a decade ago, a series of papers appeared that revealed how many classic signal processing algorithms can be regarded as message passing algorithms on FFGs (e.g., Loeliger, 2004; Dauwels et al., 2005a,b; Dauwels, 2007; Loeliger et al., 2007). More recently, FFGs have found applications in diverse subject areas such as control theory (Hoffmann and Rostalski, 2017), linear algebra (Al-Bashabsheh et al., 2011), quantum mechanics (Loeliger and Vontobel, 2017), audio processing algorithm design (Van de Laar and De Vries, 2016) and turbo equalization (Guo and Ping, 2008).

In summary, taking a place between black-box and model-specific simulation frameworks, the FFG formalism provides a visually insightful and biologically conceivable graphical process theory for describing and simulating complex active inference processes.

The goal of this paper is to introduce the FFG formalism to the systems neuroscience community. Our presentation will include development of an FFG for a deep temporal active inference (DTAI) process (Friston et al., 2017b), as this is arguably the most advanced current model for active inference. We will also develop a local-in-time-and-place message passing schedule for automated inference in DTAI models. section 2 starts with a tutorial introduction to probabilistic modeling with FFGs. section 3 proceeds with concrete graph examples for linear Gaussian dynamical systems, which are building blocks for more realistic hierarchical models of sensory observations. We follow in section 4 with FFG graphs for multi-scale hierarchical dynamical (MSHD) systems. In section 5 we show that a deep temporal active inference process is an instance of an MSHD process with a specific policy model. In particular, we address the peculiarity of having to minimize both free energy and *expected* free energy, where the latter quantity is needed to set the prior for the policies (action sequences) that are entertained by the agent. The FFG framework clearly visualizes how expected free energy minimization is nested as an inference subtask inside the full generative model. In other words, the free energy principle fully accounts for (even mandates) the minimization of expected free energy in an active inference process.

## 2. Probabilistic modeling with factor graphs

Consider a joint probability distribution *p*(*x, y, z*). Using sum and product rules we can express the conditional distribution *p*(*z*|*x*) as

$$p(z|x)=\frac{\int p(x,y,z)\text{d}y}{\iint p(x,y,z)\text{d}y\text{d}z}.$$

Since this expression is also true if *x*, *y* and *z* are vector variables, it is always possible to integrate any subset of nuisance variables out of the system and condition on any subset of observed variables. Thus, the problem of Bayesian inference is mostly a computational issue since the integral (or sum) in the denominator is often intractable. For instance, on a discrete alphabet, if *y* and *z* together contain 20 dimensions and each dimension is defined on 10 values, then the denominator contains 10^20^ terms.

### 2.1. Forney-style factor graphs

**Figure 1:**
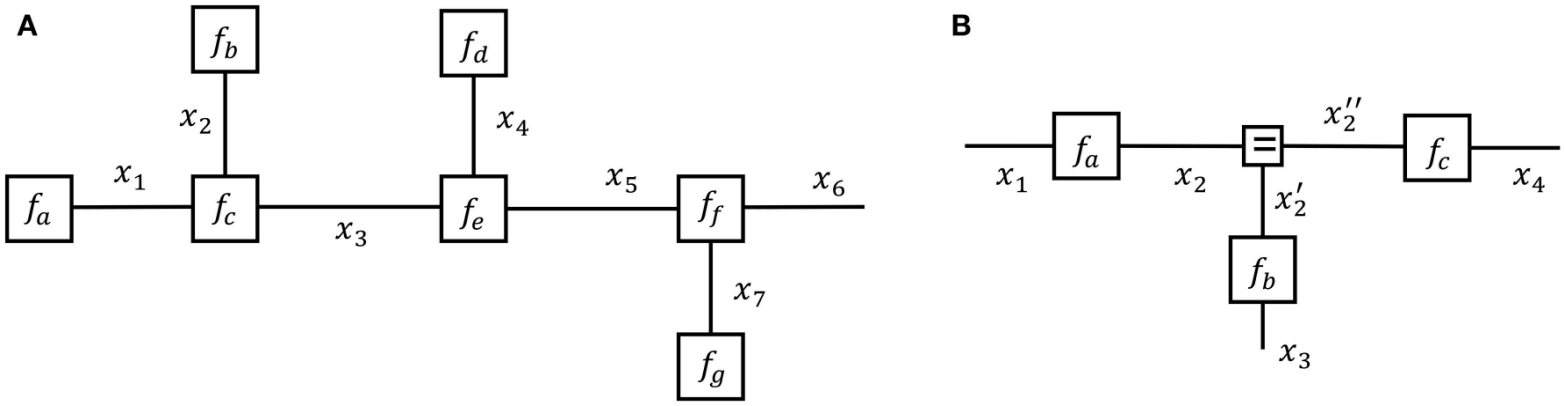
**(A)** A Forney-style factor graph representation of Equation (1). **(B)** A Forney-style factor graph for Equation (3).

The computational load of inference can be severely reduced if the model factorizes. Consider the model

(1) $$\begin{array}{l} f(x_{1},x_{2},\ldots ,x_{7})=f_{a}(x_{1})f_{b}(x_{2})f_{c}(x_{1},x_{2},x_{3})f_{d}(x_{4})f_{e}(x_{3},x_{4},x_{5})\\ \;f_{f}(x_{5},x_{6},x_{7})f_{g}(x_{7}) \end{array}$$

and the corresponding Forney-style Factor Graph (FFG) in Figure 1A. In an FFG, each factor is represented by a node and each variable by an edge. An edge attaches to a node if the edge variable is an argument of the node function. Variables that appear in only one factor (e.g., *x*_6_) are represented by a half-edge. In this paper, we will assume that both the global function *f* and factors *f*_•_ represent probability distributions.

Note that a variable name may appear in maximally two factors in an FFG, since an edge has only two end points. This would create a problem for the model

(2) $$f(x_{1},x_{2},x_{3},x_{4})=f_{a}(x_{1},x_{2})f_{b}(x_{2},x_{3})f_{c}(x_{2},x_{4}),$$

since *x*_2_ appears in three factors. This issue can be solved by extending the model to

(3) $$g(x_{1},x_{2},x_{2}^{\prime },x_{2}^{\prime \prime },x_{3},x_{4})=f_{a}(x_{1},x_{2})f_{b}(x_{2}^{\prime },x_{3})f_{c}(x_{2}^{\prime \prime },x_{4})f_{d}(x_{2},x_{2}^{\prime },x_{2}^{\prime \prime }),$$

where $f_{d}\left(x_{2},x_{2}^{\prime },x_{2}^{\prime \prime }\right)≜\delta \left(x_{2}-x_{2}^{\prime }\right)\delta \left(x_{2}-x_{2}^{\prime \prime }\right)$. The node *f*_*d*_ is called an *equality* (or *branching*) node and the corresponding FFG for *g* is shown in Figure 1B. Note that each variable in *g* appears in maximally two factors through the introduction of two auxiliary variables $x_{2}^{\prime }$ and $x_{2}^{\prime \prime }$. Since *f* is a marginal of *g*, i.e.,

$$f(x_{1},x_{2},x_{3},x_{4})=\int g(x_{1},x_{2},x_{2}^{\prime },x_{2}^{\prime \prime },x_{3},x_{4})\text{d}x_{2}^{\prime }\text{d}x_{2}^{\prime \prime },$$

any inference problem on *f* can be executed by a corresponding inference problem on *g*. Equality nodes make it possible to draw an appropriate FFG for any factorized probability distribution, regardless of the number of factors that share a particular variable.

### 2.2. Inference by message passing in a factor graph

For the model given by Equation (1), assume that we are interested in the marginal

(4) $$\bar{f}(x_{3})=\;\int \ldots \int f(x_{1},x_{2},\ldots ,x_{7})\text{d}x_{1}\text{d}x_{2}\text{d}x_{4}\text{d}x_{5}\text{d}x_{6}\text{d}x_{7}.$$

Due to the factorization, we can decompose this sum by the distributive law as

(5) $$\begin{array}{l} \bar{f}(x_{3})=\underset{{\vec{\mu }}_{X_{3}}(x_{3})}{\underset{︸}{\left(\iint f_{a}(x_{1})f_{b}(x_{2})f_{c}(x_{1},x_{2},x_{3})\text{d}x_{1}\text{d}x_{2}\right)}}\\ \;\times \underset{{\overset{\leftarrow }{\mu }}_{X_{3}}(x_{3})}{\underset{︸}{\left(\iint f_{e}(x_{3},x_{4},x_{5})\cdot \overset{{\vec{\mu }}_{X_{4}}(x_{4})}{\overset{︷}{f_{d}(x_{4})}}\cdot \underset{{\overset{\leftarrow }{\mu }}_{X_{5}}(x_{5})}{\underset{︸}{\left(\iint f_{f}(x_{5},x_{6},x_{7})\overset{{\overset{\leftarrow }{\mu }}_{X_{7}}(x_{7})}{\overset{︷}{f_{g}(x_{7})}}\text{d}x_{6}\text{d}x_{7}\right)}}\text{d}x_{4}\text{d}x_{5}\right)}} \end{array}$$

which contains (far) fewer computations than the full 6-dimensional integral of Equation (4).

**Figure 2:**
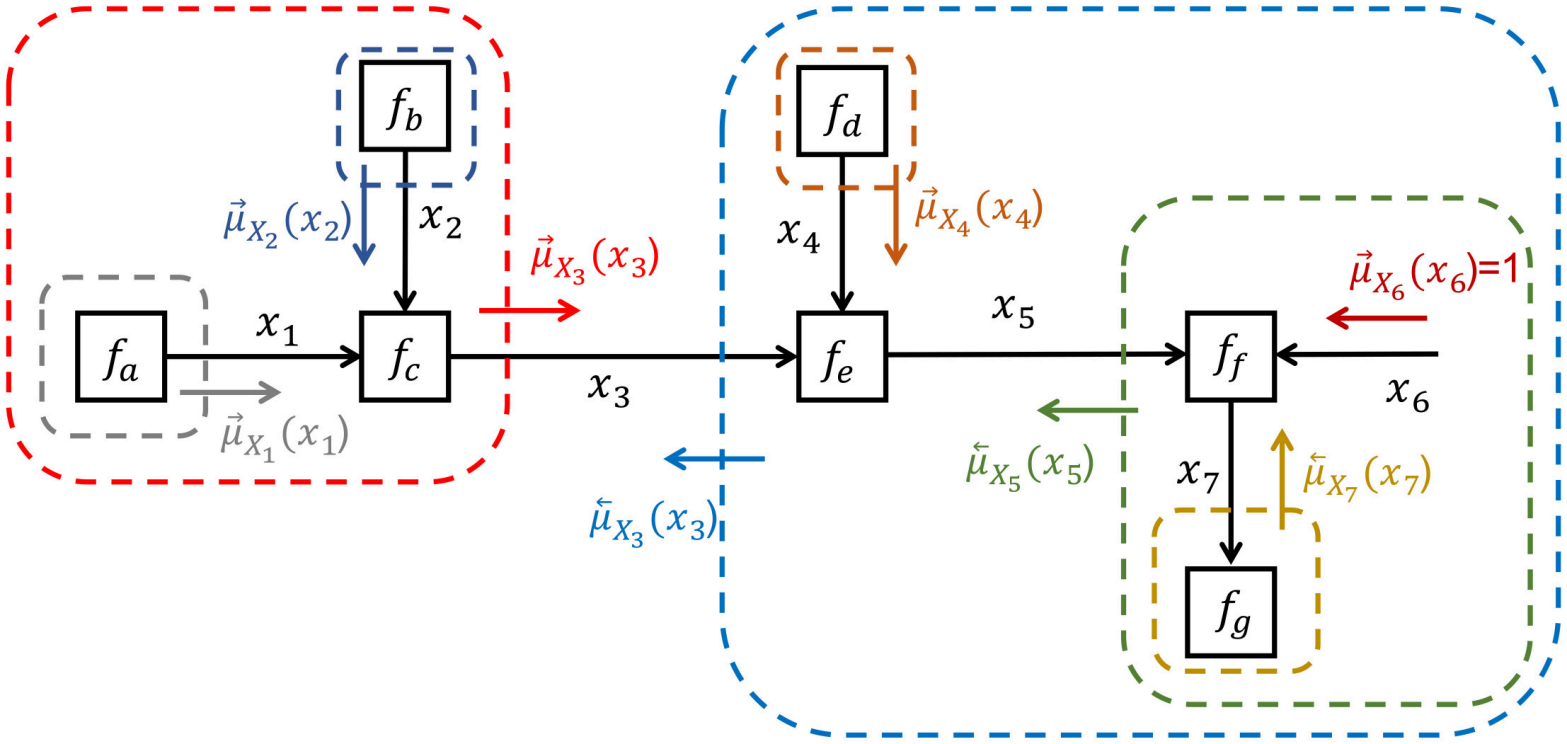
Inference by sum-product message passing for model Equation (1).

In order to distinguish between a *forward* message ${\vec{\mu }}_{X_{3}}\left(x_{3}\right)$ and a *backward* message ${\overset{⃖}{\mu }}_{X_{3}}\left(x_{3}\right)$, it can be useful to draw the graph with *directed* arrows as in Figure 2. Principally though, an FFG is an undirected graph and the direction of arrows has no computational consequences.

The message ${\vec{\mu }}_{X_{3}}\left(x_{3}\right)$ is obtained by multiplying all factors inside the red box (in Figure 2), followed by marginalization over all variables whose edges are fully inside the box, i.e.,

(6) $${\vec{\mu }}_{X_{3}}(x_{3})=\iint f_{a}(x_{1})f_{b}(x_{2})f_{c}(x_{1},x_{2},x_{3})\text{d}x_{1}\text{d}x_{2}.$$

The result of this *closing-the-box* procedure is a function of the variables whose edges cross the border of the box, which in this case is *x*_3_. This function ${\vec{\mu }}_{X_{3}}\left(x_{3}\right)$ is called a *sum-product* message and rather than drawing boxes in the FFG, it is customary to draw a message by a small arrow next to the edge. If the global function *f* is a joint probability distribution, then message ${\vec{\mu }}_{X_{3}}\left(x_{3}\right)$ represents a probability distribution over *x*_3_ that encodes the beliefs about *x*_3_ that is contained inside the red box. Similarly, closing the blue box around nodes *f*_*d*_ through *f*_*g*_ leads to message ${\overset{⃖}{\mu }}_{X_{3}}\left(x_{3}\right)$, which hold the beliefs about *x*_3_ that is contained in the subgraph to the right of the *x*_3_-edge. The marginal for *x*_3_ is obtained by multiplying forward and backward messages, i.e., $\overset{-}{f}\left(x_{3}\right)={\vec{\mu }}_{X_{3}}\left(x_{3}\right){\overset{⃖}{\mu }}_{X_{3}}\left(x_{3}\right)$. This multiplication fuses information about *x*_3_ from the sub-graphs to the left and right sides of the *x*_3_-edge.

**Figure 3:**
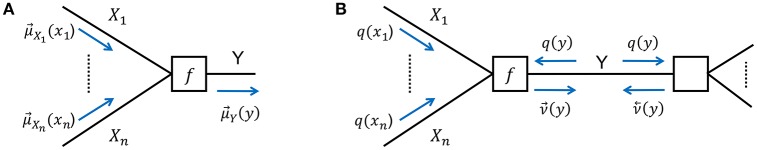
**(A)** The sum-product update rule of Equation (7). **(B)** The variational update rule, see Equation (12).

The closing-the-box procedure can be nested until each box contains one node with (possibly multiple) incoming messages and one outgoing message, e.g., see the nesting in Figure 2 to compute ${\overset{⃖}{\mu }}_{X_{3}}\left(x_{3}\right)$. Thus, the marginal $\overset{-}{f}\left(x_{3}\right)$ can be inferred in the FFG by passing sum-product messages from the terminal nodes toward *x*_3_. In this view, each node just processes locally incoming messages to produce an outgoing message. The *sum-product update rule* for a node is simply the product of all incoming messages with the factor, followed by marginalization over the variables on the incoming edges, see Figure 3A. This is a message passing-based rewrite of the closing-the-box rule. For instance, the sum-product update rule for ${\overset{⃖}{\mu }}_{X_{3}}\left(x_{3}\right)$ can be written as (see also Figure 2)

(7) $${\overset{\leftarrow }{\mu }}_{X_{3}}(x_{3})=\iint f_{e}(x_{3},x_{4},x_{5}){\vec{\mu }}_{X_{4}}(x_{4}){\overset{\leftarrow }{\mu }}_{X_{5}}(x_{5})\text{d}x_{4}\text{d}x_{5}.$$

It is easy to verify that applying the closing-the-box rule to a terminal node yields the factor itself as the sum-product message, e.g., ${\overset{⃖}{\mu }}_{X_{7}}\left(x_{7}\right)=f_{g}\left(x_{7}\right)$. The incoming message from a half-edge (e.g., *x*_6_) is always equal to 1. This can simply be checked by realizing that adding a terminal node *f*_*h*_(*x*_6_) = 1 to the graph would not change the global function. In other words, the incoming message from a half-edge is uninformative.

In order to compute all marginals (for all edges) in a graph, we start with incoming messages at the terminals and half-edges, and proceed until each edge has both forward and backward messages. The *sum-product theorem* states that, if the graph is a tree, then multiplication of the forward and backward messages on an edge yields the exact (Bayesian) marginal for the corresponding variable (Kschischang et al., 2001). If the graph contains cycles, then this result cannot be guaranteed. In practice, good approximate inference results are often obtained in cyclic graphs by iterative message passing updating, as if the cyclic graph were unrolled as a deep tree (Vasudeva Raju and Pitkow, 2016). The literature also uses the term *belief propagation* to describe inference approximation by iterative sum-product message passing on general graphs (the term was originally introduced by Pearl, 1982).

Application of the closing-the-box procedure around a set of nodes yields a new “composite” node that hides the internal structure of the box without effect on the interfaces with the rest of the graph. This is a very attractive property of FFGs that provides a hierarchical view on network structures. For instance, in Figure 2, closing the red box around *f*_*a*_, *f*_*b*_ and *f*_*c*_ yields a new node *f*_*abc*_(*x*_3_) with factor (and outgoing message) given by Equation (1). Application of the closing-the-box procedure around an entire graph yields a number (not a function of a variable). This number equals the Bayesian evidence for that graph.

There is an interesting interpretation of message passing that sits well with the surprise minimization view of the free-energy principle. The outgoing message of a node is (proportional to) the posterior probability distribution for the outgoing edge variable, after all information in the box has been processed by marginalization. It is easy to convert a probability distribution *p*(*x*) for variable *x* to the *surprise* (or self-information) *I*(*x*) ≜ − log*p*(*x*) for that variable. Thus, taking a surprise point of view, node processing is an effort to explain away incoming surprise and pass on the remaining surprise in outgoing messages. When there is no surprise left, there is nothing to communicate and messages become uninformative. In other words, message passing is a distributed surprise minimization process.

### 2.3. Variational message passing

Inference by sum-product message passing works great in many factor graphs and we will go through an illustrative example (Kalman filtering) in section 3.2. Still, sum-product message passing is not always appropriate. For instance, the sum over all settings for discretely valued variables may have too many terms to be computable in the time available. If the hidden variables are continuously valued, then the sum-product update rule may not lead to an analytical expression. Moreover, on loopy graphs the product of forward and backward messages does not lead to the true (Bayesian) marginal. These or similar computational issues are shared by any (biological or artificial) agent that attempts to execute exact Bayesian inference. The free energy principle asserts that real brains cope with these computational issues by instead minimizing a free energy functional, which effectively transforms an intractable inference problem to a tractable (approximately correct) optimization problem.

Consider a model *p*(*z, o*), where *o* collects all observations and *z* contains all hidden variables in the system, including states *s*, controls *u* and parameters θ^1^. The goal of Bayesian inference is to compute the latent variable posterior *p*(*z*|*o*) and the model evidence *p*(*o*). Rather than computing *p*(*z*|*o*) precisely, we consider an approximate solution *q*(*z*) that is known as the *proposal* (or *recognition*) distribution. The (Gibbs) free-energy (FE) functional is defined as

(8) $$F[q]≜\int q(z)\text{log}\frac{q(z)}{p(z,o)}\text{d}z.$$

Making use of *p*(*z, o*) = *p*(*z*|*o*)*p*(*o*), it is easy to decompose *F* as

(9) $$F[q]=\underset{surprise}{\underset{︸}{-\text{log }p(o)}}+\underset{divergence}{\underset{︸}{\text{D}\left(q(z)\|p(z|o)\right)}},$$

where $\text{D}\left(q\left(x\right)||p\left(x\right)\right)≜\underset{x}{\sum }q\left(x\right)\text{log}\frac{q\left(x\right)}{p\left(x\right)}$ is known as the *Kullback-Leibler* (KL) divergence between distributions *q* and *p*. When *p* and *q* are exactly the same functions, the KL divergence equals zero and in all other cases the KL divergence is greater than zero. Therefore, the FE is an upper bound to the surprise (or equivalently, a lower bound to the logarithm of model evidence log*p*(*o*)). Minimization of *F* with respect to *q* leads to an approximate posterior q^*^(*z*) ≈ *p*(*z*|*o*) and an approximate (negative log) evidence estimate *F*[q^*^] ≈ − log*p*(*o*).

A common approach to free energy minimization starts with an assumed factorization of the proposal distribution as

(10) $$q(z)=\prod_{i=1}^{m}q_{i}(z_{i})$$

where *z* = (*z*_1_, …, *z*_*m*_) is a partitioning of the hidden variables into disjoint sets. This factorization is known as the *mean field* assumption. In that case, the minimizing solutions obey the following relation (for all *i*) (Bishop, 2006):

(11) $$\begin{array}{l} \text{log }q_{i}^{*}(z_{i})\propto E_{q_{j\neq i}^{*}}\left[\text{log }p(z_{1},\ldots ,z_{m},o)\right]\\ \;≜\int \ldots \int q_{1}^{*}(z_{1})\ldots q_{i-1}^{*}(z_{i-1})q_{i+1}^{*}(z_{i+1})\ldots q^{*}(z_{m})\\ \;\cdot \text{log }p(z_{1},\ldots ,z_{m},o)\text{d}z_{1}\ldots \text{d}z_{i-1}\text{d}z_{i+1}\ldots \text{d}z_{m}. \end{array}$$

Variational inference proceeds by iteratively executing Equation (11), where current best estimates for $q_{j}^{*}\left(z_{j}\right)$ are substituted where needed (see e.g., Bishop, 2006). In case the generative model factorizes, Equation (11) provides the basis for a message passing realization of variational inference.

Consider Figure 3B with factor *f*(*x*_1_, …, *x*_*n*_, *y*). *Variational message passing* (VMP) for edge *Y* proceeds by a two-step recipe: first, given incoming (marginal) messages *q*_*i*_(*x*_*i*_) for *i* = 1, …, *n*, an outgoing (variational) message $\vec{\nu }\left(y\right)$ is computed by

(12) $$\vec{\nu }(y)\propto \text{exp}\left({\text{E}}_{q_{i}}\left[\text{log }f(x_{1},\ldots ,x_{m},y)\right]\right),$$

and similarly for backward message $\overset{⃖}{\nu }\left(y\right)$. This is followed by updating the marginal $q\left(y\right)\propto \vec{\nu }\left(y\right)\cdot \overset{⃖}{\nu }\left(y\right)$ and sending the marginal back to the two nodes that connect to *Y*. Next, the messages and marginal for another edge are updated, e.g., for one of the *X*_*i*_-labeled edges, see Dauwels (2007) for a detailed description of VMP on FFGs.

Equation (8) is not the only possible free energy functional. Minka (2005) discusses a large family of information divergence-based loss functions that lead to alternative message passing algorithms. In fact, sum-product message passing can also be derived from minimizing the so-called Bethe free energy (Yedidia et al., 2005). There is no principal reason against combining different message update rules for different edges in a graph, e.g., sum-product message passing can easily be combined with variational message passing (Riegler et al., 2013).

In summary, Forney-style factor graphs provide both a visually insightful representation and a powerful computational process theory for minimizing free energy functionals of probabilistic models by message passing.

## 3. Linear dynamical systems and Kalman filtering

Forney-style factor graphs are particularly useful to automate inference by message passing in dynamical systems. In this section we describe the FFG and message passing inference for a simple linear Gaussian dynamical system, which is an important building block for more complex structures.

### 3.1. Model specification

**Figure 4:**
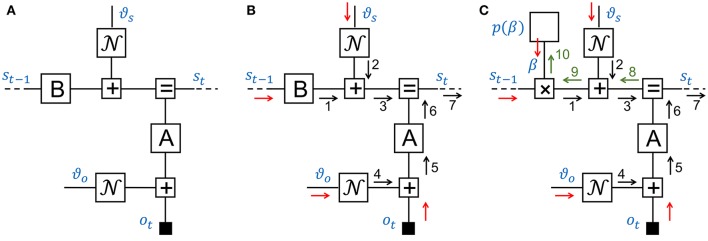
**(A)** A Forney-style factor graph for one time step of a linear Gaussian dynamical system. **(B)** A 7-step sum-product message passing sequence for Kalman filtering. **(C)** Three additional messages (8–10) afford learning the state transition gain *β* from observations.

A Linear Gaussian Dynamical System (LGDS) is described by

(13a) $$\overset{\text{generative model}}{\overset{︷}{p(s_{0},o_{1},s_{1},\ldots ,o_{n},s_{n})}}=\overset{\text{prior}}{\overset{︷}{p(s_{0})}}\prod_{t=1}^{n}\overset{\begin{array}{c} \text{state transition}\\ \text{model} \end{array}}{\overset{︷}{p(s_{t}|s_{t-1})}}\overset{\begin{array}{c} \text{observation}\\ \text{model} \end{array}}{\overset{︷}{p(o_{t}|s_{t})}}$$

(13b) $$p(s_{t}|s_{t-1})=N\left(s_{t}|Bs_{t-1},\vartheta_{s}\right)$$

(13c) $$p(o_{t}|s_{t})\;=N\left(o_{t}|As_{t},\vartheta_{o}\right)$$

where N(x|μ,ϑ) indicates a (possibly multivariate) Gaussian distribution over *x* with mean μ and variance (matrix) ϑ. This model describes how observations *o*_*t*_ for *t* = 1, …, *n* are generated by a dynamical system with unobserved states *s*_*t*_ and parameters θ = {*A, B*, ϑ_*s*_, ϑ_*o*_}. The FFG for this system is displayed in Figure 4A. Note that the graph only shows one time step and dashed line segments to the left and right of the segment indicate that the graph extends in the same way to both sides.

### 3.2. Kalman filtering by message passing

Consider the inference task of updating the hidden state estimate at time step *t*, based on a given state estimate at *t*−1 and a new observation *o*_*t*_. The name *Kalman filtering* relates to an efficient recursive algorithm to solve this inference problem (Kalman, 1960). Kalman filtering represents a cornerstone of state-space modeling-based engineering fields such as signal processing and control theory as well as serves as a basic dynamic model for human perception. Since the joint distribution of observed and latent variables in a LGDS is multivariate Gaussian, the (Kalman) state estimate *s*_*t*_ given observations *o*_1:*t*_ is necessarily also described by a Gaussian distribution. Much of the dynamic systems literature is devoted to extending the Kalman filter to deal with more relaxed model assumptions including non-linearities, non-Gaussian disturbances and hierarchical structures. Figure 4B shows the FFG and a sum-product message passing sequence for the Kalman filter. Note that the edge for *o*_*t*_ is now terminated by a (small) black node to indicate that *o*_*t*_ is observed. If *o*_*t*_ were unobserved, the corresponding half-edge would pass an uninformative message $\overset{⃖}{\mu }\left(o_{t}\right)=1$ into the graph. If *o*_*t*_ is observed, say *o*_*t*_ = ô_*t*_, then the black node sends a delta message $\overset{⃖}{\mu }\left(o_{t}\right)=\delta \left(o_{t}-{ô}_{t}\right)$ into the graph. The graph can be viewed as a tree below the “root node” *s*_*t*_. The update for *s*_*t*_ is contained in message 7, which can be computed by a message passing sequence that starts at the terminals of the tree (i.e., at *s*_*t*−1_, ϑ_*s*_, ϑ_*o*_, *o*_*t*_) and moves up the tree toward message 7 (see Figure 4B).

Let us work out the sum-product update rules for a few messages. Message 3 is the outgoing message of an addition node with factor *f*(*x, y, z*) = δ(*x* + *y* − *z*), see also Table 1, row 1. The outgoing message in the direction of *z* is given by

(14) $$\begin{array}{l} {\vec{\mu }}_{Z}(z)=\int \delta (x+y-z){\vec{\mu }}_{X}(x){\vec{\mu }}_{Y}(y)\text{d}x\text{d}y\\ \;=\int {\vec{\mu }}_{X}(z){\vec{\mu }}_{Y}(z-x)\text{d}x, \end{array}$$

**Table 1:** Sum-product update rules some standard nodes with Gaussian messages, see Korl (2005, ch. 4) and Loeliger et al. (2007) for more elaborate tables.

**#No**	**Node**	**Factor update rule**
1	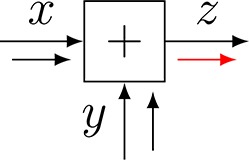	$\begin{array}{l} \;f(x,y,z)=\delta (x+y-z)\\ m_{z}=m_{x}+m_{y}\\ \vartheta_{z}=\vartheta_{x}+\vartheta_{y} \end{array}$
	Addition
2	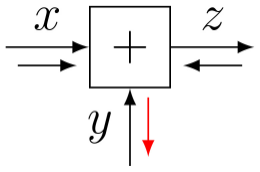	$\begin{array}{l} f(x,y,z)=\delta (x+y-z)\\ m_{y}=m_{z}-m_{x}\\ \vartheta_{y}=\vartheta_{z}+\vartheta_{x} \end{array}$
	Subtraction
3	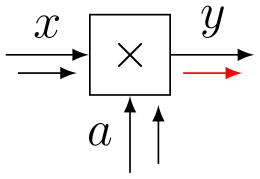	$\begin{array}{l} f(x,y,a)=\delta (y-ax)\\ m_{y}=am_{x}\\ \vartheta_{y}=a^{2}\vartheta_{x} \end{array}$
	Multiplication (forward)
4	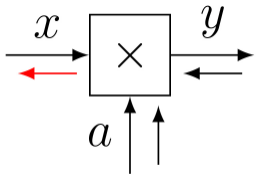	$\begin{array}{l} f(x,y,a)=\delta (y-ax),a\neq 0\\ m_{x}=a^{-1}m_{y}\\ \vartheta_{x}=a^{-2}\vartheta_{y} \end{array}$
	Multiplication (backward)
5	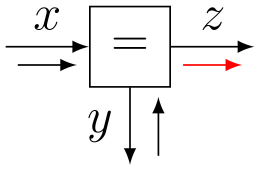	$\begin{array}{l} f(x,y,z)=\delta (x-y)\delta (x-z)\\ m_{z}=\vartheta_{z}(\vartheta_{x}^{-1}m_{x}+\vartheta_{y}^{-1}m_{y})\\ \vartheta_{z}=\vartheta_{x}\vartheta_{y}{\left(\vartheta_{x}+\vartheta_{y}\right)}^{-1} \end{array}$
	Equality
6	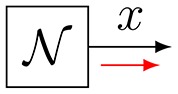	$\begin{array}{l} f(x)=N(m,\vartheta )\\ m_{x}=m\\ \vartheta_{x}=\vartheta \end{array}$
	Gaussian

i.e., ${\vec{\mu }}_{Z}\left(z\right)$ is the convolution of messages ${\vec{\mu }}_{X}\left(x\right)$ and ${\vec{\mu }}_{Y}\left(y\right)$. For Gaussian incoming messages ${\vec{\mu }}_{X}\left(x\right)=N\left(x|m_{x},\nu_{x}\right)$ and ${\vec{\mu }}_{Y}\left(y\right)=N\left(y|m_{y},\nu_{y}\right)$, Equation (14) evaluates to ${\vec{\mu }}_{Z}\left(z\right)=N\left(z|m_{x}+m_{y},\nu_{x}+\nu_{y}\right)$. This makes sense, since the means and variances add for the sum of two (uncorrelated) variables.

Next, we consider the outgoing message 7 of an equality node *f*(*x, y, z*) = δ(*x* − *y*)δ(*x* − *z*), see also Table 1, row 5 and message 7 in Figure 4B. The outgoing sum-product message is given by

(15) $$\begin{array}{l} {\vec{\mu }}_{Z}(z)=\int \delta (x-y)\delta (x-z){\vec{\mu }}_{X}(x){\vec{\mu }}_{Y}(y)\text{d}x\text{d}y\\ \;={\vec{\mu }}_{X}(z)\int \delta (z-y){\vec{\mu }}_{Y}(y)\text{d}y\\ \;={\vec{\mu }}_{X}(z){\vec{\mu }}_{Y}(z). \end{array}$$

Hence, the outgoing message of an equality node involves multiplication of the incoming messages. For Gaussian input messages, this implies that the outgoing message is also a Gaussian message with added precision-weighted means and added precisions of incoming messages. Apparently, the equality node in Figure 4B serves to implement Bayes rule. Incoming message 3 can be interpreted as a prior-based state prediction message and message 6 as a likelihood message that processes *o*_*t*_. Message 7 fuses information from the prior predictive and likelihood messages by Bayes rule. For a full description of Kalman filtering by sum-product message passing, see Loeliger et al. (2007).

For a large range of simple node functions, it is possible to derive analytical sum-product update rules. Table 1 shows a small sample of these rules for common factors. In a computer simulation context, if these rules are stored in a lookup table, then inference can be automatically executed in freely definable graphs. It can also be useful to tabulate the messages for certain composite nodes, e.g., by closing a box around the equality node and the likelihood factor *A* in Figure 4A. Rather than passing messages inside the composite node, it may be computationally advantageous to compute the messages (going out of the composite node) by a custom algorithm. Using this method, Loeliger et al. (2007) and Loeliger et al. (2016) present message update rules for composite nodes that facilitate Kalman filtering with improved numerical stability and computational load.

### 3.3. Dynamical systems with control signals

We now consider an extension of the LGDS model where the state transition model can be controlled by another agent. This feature will be important when we consider hierarchical systems. To keep it simple, we assume that the state transition model is given by

(16a) $$p(\beta )=N(\beta |m_{\beta },\vartheta_{\beta })$$

(16b) $$p(s_{t}|s_{t-1})=N(s_{t}|\beta s_{t-1},\vartheta_{s})$$

where *β* is a scalar gain, see Figure 4C. From the viewpoint of the original LGDS (without prior for *β*), *β* can be interpreted as a external control signal that affects the state transition model and the prior *p*(*β*) can be viewed as a model for the controller. The controller node may comprise a large network that is contained in a composite node *p*(*β*) in Figure 4C. Assume that we are interested in learning an appropriate controller from observations. This would involve extending the Kalman filtering message sequence by messages 8–10 in Figure 4C. Message 10 comprises new information about *β* that is obtained from observation *o*_*t*_. In **Appendix A**, we derive a Gaussian variational message for message 10.

## 4. Hierarchical dynamical systems

Natural signals are hierarchically organized. For instance, speech signals contain patterns over multiple time scales including sentences (~1 s), phonemes (~10^−1^ s), glottal pulses (~10^−2^ s) and formants (~10^−3^ s) (Turner and Sahani, 2008). As a consequence, active inference processes in the brain rely on multi-scale hierarchical dynamical (MSHD) models. In this section we consider an FFG description of an MSHD model that was originally presented to demonstrate a deep temporal model for reading (Friston et al., 2017b)^2^. Our purpose here is solely to describe how the FFG framework provides insights to both the model definition and inference issues.

### 4.1. Model specification

**Figure 5:**
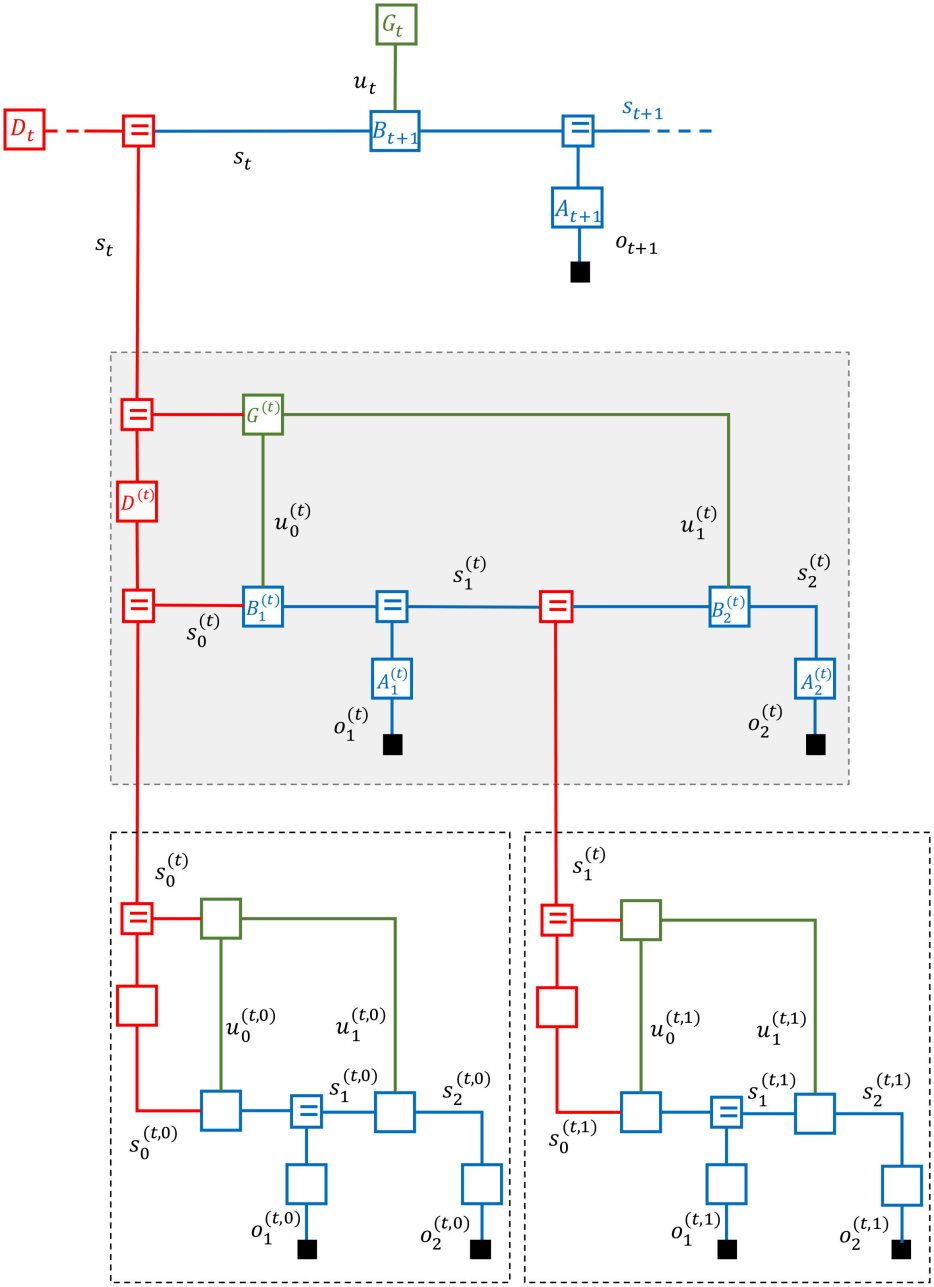
A Forney-style factor graph of a three-layer multi-scale hierarchical dynamical system.

Consider the three-layer MSHD system in Figure 5. The FFG displays one time step for the top (third) layer with generative model

(17) $$\underset{A_{t+1}}{\underset{︸}{p(o_{t+1}|s_{t+1})}}\underset{B_{t+1}}{\underset{︸}{p(s_{t+1}|s_{t},u_{t})}}\underset{D_{t}}{\underset{︸}{p(s_{t})}}\underset{G_{t}}{\underset{︸}{p(u_{t})}}.$$

In this equation, *A*_*t*_ represents the likelihood model, *B*_*t*_ refers to the state transition model, *G*_*t*_ stands for the prior on the control signal *u*_*t*_ and *D*_*t*_ serves as the state prior. If *o*_*t*+1_ is observed (indicated in the figure by the solid rectangle that terminates the *o*_*t*+1_ edge), then the state *s*_*t*+1_ gets updated by a likelihood message emanating from *o*_*t*+1_. Additionally, during time step *t*, the top layer receives a second likelihood message from lower levels (over the red edge with label *s*_*t*_).

In order to enhance the visual interpretation of the state space models in Figure 5, we have grouped the state transition *B* and additive state noise model $N\left(0,\vartheta_{s}\right)$ of Figure 4 into a single composite state transition node *B*_*t*_ (and similarly for the likelihood and observation noise models). Note that the entire circuit comprising first and second layers can also be viewed as a composite likelihood node for the top layer.

In this example, the middle layer takes two time steps of a state-space model during one time step for the top layer. In general, lower levels may take multiple time steps during a single step for the top layer. In Figure 5, the middle layer is outlined by a shaded box.

The generative model for the second layer is given by

(18) $$\overset{\text{priors}}{\overset{︷}{\underset{D^{\left(t\right)}}{\underset{︸}{p(s_{0}^{\left(t\right)}|s_{t})}}\underset{G^{\left(t\right)}}{\underset{︸}{p(u_{0}^{\left(t\right)},u_{1}^{\left(t\right)}|s_{t})}}}}\overset{\text{state-space model}}{\overset{︷}{\prod_{k=1}^{2}\underset{A_{k}^{\left(t\right)}}{\underset{︸}{p(o_{k}^{\left(t\right)}|s_{k}^{\left(t\right)})}}\underset{B_{k}^{\left(t\right)}}{\underset{︸}{p(s_{k}^{\left(t\right)}|s_{k-1}^{\left(t\right)},u_{k-1}^{\left(t\right)})}}}}$$

The superscript (*t*) indicates the context, namely, the time step *t* of the superior layer. Crucially, both the prior of the initial state and control signals are now dependent on the current state *s*_*t*_ of the superior layer. Since the middle layer takes two steps, there are two control signals $u_{0}^{\left(t\right)}$ and $u_{1}^{\left(t\right)}$. It is common parlance to denote a sequence of (future) control signals by a *policy* $\pi^{\left(t\right)}≜\left(u_{0}^{\left(t\right)},u_{1}^{\left(t\right)}\right)$, In other words, rather than the control prior $p\left(u_{0}^{\left(t\right)},u_{1}^{\left(t\right)}|s_{t}\right)$, we could have written a policy prior $p\left(\pi^{\left(t\right)}|s_{t}\right)$. Note that both the top and middle layers are by themselves regular dynamical systems as described in section 3. The additional information in the hierarchical model lies in the specification of how the (initial state and policy) priors of a layer depend on the current state of the superior layer.

In similar fashion, at each time step, we allow the state of the middle layer to generate both an “in-layer” observation and observations at lower layers. In Figure 5, we have again assumed two time steps for the first (bottom) layer during execution of one step of the second layer. To be precise, the dynamics of the first layer are given by

(19) $$\overset{\text{initial state and policy priors}}{\overset{︷}{\prod_{k=0}^{1}\underset{D^{\left(t,k\right)}}{\underset{︸}{p(s_{0}^{\left(t,k\right)}|s_{k}^{\left(t\right)})}}\underset{G^{\left(t,k\right)}}{\underset{︸}{p(u_{0}^{\left(t,k\right)},u_{1}^{\left(t,k\right)}|s_{k}^{\left(t\right)})}}}}\overset{\text{state-space model}}{\overset{︷}{\prod_{n=1}^{2}\underset{A_{n}^{\left(t,k\right)}}{\underset{︸}{p(o_{n}^{\left(t,k\right)}|s_{n}^{\left(t,k\right)})}}\underset{B_{n}^{\left(t,k\right)}}{\underset{︸}{p(s_{n}^{\left(t,k\right)}|s_{n-1}^{\left(t,k\right)},u_{n-1}^{\left(t,k\right)})}}}}.$$

The context for the first layer states is uniquely described by the tuple (*t, k*), where *t* and *k* are the current time steps for the top and middle layers, respectively.

The full generative model for one step of the top layer is specified by the multiplication of Equations (17–19).

While this set of equations comprises an exact specification of the generative model, the notational overhead for keeping track of context in superscripts in hierarchical models is rather cumbersome. This is another reason why the graphical FFG notation for hierarchical models is preferred.

### 4.2. Inference

**Figure 6:**
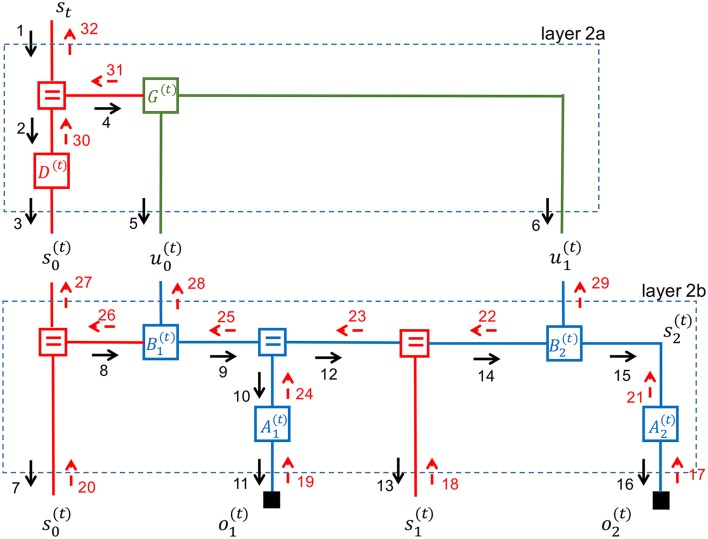
Message passing sequence in a multi-scale hierarchical dynamical system.

We now turn attention to inference in the MSHD model. The inference objective at time step *t* is to update the beliefs about all hidden variables in the graph, based on all observations $\left(o_{t+1},o_{1}^{\left(t\right)},o_{2}^{\left(t\right)},o_{1}^{\left(t,0\right)},o_{2}^{\left(t,0\right)},o_{1}^{\left(t,1\right)},o_{2}^{\left(t,1\right)}\right)$ (all solid black rectangles). The update process is steered by the goal to minimize free energy in the graph and can be executed through message passing. Various message passing sequence schedules and update rules are possible, but all schemes rely on top-down prediction passes followed by bottom-up correction steps. A possible message passing schedule for the middle layer is shown in Figure 6.

Note that the graph for the middle layer contains loops. This is a problem since the product of a backward and forward sum-product message is no longer guaranteed to be equal to the marginal distribution. In practice, multiple iterations of updates over each edge may be necessary to converge to a good-enough approximate inference process.

Forney-style factor graphs make it easy to visualize how such iterative updating schedules work. In Figure 6, we have partitioned the graph for the middle layer into two simple (i.e., non-loopy) subgraphs. Inside these subgraphs, which are labeled layer-2a and layer-2b, correct marginals can be obtained by one forward-backward pass. The inference procedure begins by partitioning the full graph into simple subgraphs. Each simple subgraph processes incoming evidence autonomously. Subgraphs first update the marginals of hidden variables whenever new evidence is presented through incoming messages at their terminals, and follow up by updating outgoing messages.

Let us consider this procedure for the middle layer in Figure 6. We assume that the top layer sends a message (labeled 1) that contains a belief for the top layer state *s*_*t*_. Layer 2a will now update the marginals for its hidden states and pass on the effects of the new evidence to its terminals. We can ignore backward messages since there is no incoming evidence yet from layer 2b. Layer 2a will propagate the effect of message 1 to layer 2b by a forward pass that results in messages 3, 5, and 6.

Layer 2b has now new evidence coming in from layer 2a and no new evidence from lower layers. Similarly to layer 2a, layer 2b will process the incoming messages by a forward (prediction) pass that results in outgoing messages 7, 11, 13, and 16.

Layer 2b will now wait until corrective evidence is passed into its bottom terminals through messages 17–20. Next, the internal marginals are updated in Layer 2b through messages 21-26 and any remaining free energy is passed up to layer 2a through messages 27–29. In turn, layer 2a updates its internal states by messages 30–31 and unexplained evidence is passed up to layer 1 through message 32.

Thus, at the full graph level, inference proceeds by a forward layer-by-layer prediction pass, followed by a corrective backward pass based on evidence that is collected at each layer. Note that after layer 2b has updated the marginals for its internal states based on incoming evidence from layer 1, it could in principle update outgoing messages toward both layer 2a and back to layer 1. In other words, it is possible to iterate updates between two sub-layers (say, layers 1 and 2a) before sending the results to other parts of the graph. There exists little theory about what is the best scheduling strategy here. It is an unexplored but intriguing thought to treat this “inference scheduling” issue itself as the solution to a policy inference process that is subject to the free-energy principle.

## 5. Deep temporal active inference

In this section we extend the multi-scale hierarchical dynamical system to describe the deep temporal active inference (DTAI) process as discussed in Friston et al. (2017b), who illustrate a three-layer active inference process by a reading example. The task of the top layer is to infer which sentence (out of a finite set) is being read. Each sentence is formed by a sequence of words and each word consists of a sequence of letters. The middle and bottom layers infer which word and letter are currently being read, respectively. The middle and bottom layers also get to select (muscle) actions to control where the eye will look next to read a word or letter. Both perception (decoding letters, words and sentences) and actions are inferred by free energy minimization. Friston et al. (2017b) shows that this recipe leads to actions that seek to maximize information gain as the eye keeps moving to letter (and word) locations where it expects to resolve the most uncertainty about the current word (and sentence). Next, we present an FFG description of such deep temporal active inference processes.

### 5.1. Model specification

In the dynamic systems that we have described, state transitions can be modified by control signals. In MSHD systems, these control signals are a function of the state of the superior layer. We will follow the control theory nomenclature where a sequence of (future) control signals is called a *policy*. For example, the *policy model* in layer 2 of the MSHD system of section 4 is specified by $p\left(\pi^{\left(t\right)}|s_{t}\right)$, where $\pi^{\left(t\right)}≜\left(u_{0}^{\left(t\right)},u_{1}^{\left(t\right)}\right)$ is the policy at step *t*.

How should we choose policy models? In a reinforcement learning setting, it is common to specify a “reward” function and select policies that aim to maximize expected future rewards. In contrast, active inference systems, including its policy models, submit to the free energy principle and consequently make no use of externally defined reward functions. Rather, the only information that an active inference process uses is the self-knowledge that it will minimize free energy in the future. Consequently, the sole self-consistent policy selection process is to choose controls that minimize expected future free energy. Thus, a deep temporal active inference model is an instance of an MSHD system with *expected* free energy as its policy model.

**Figure 7:**
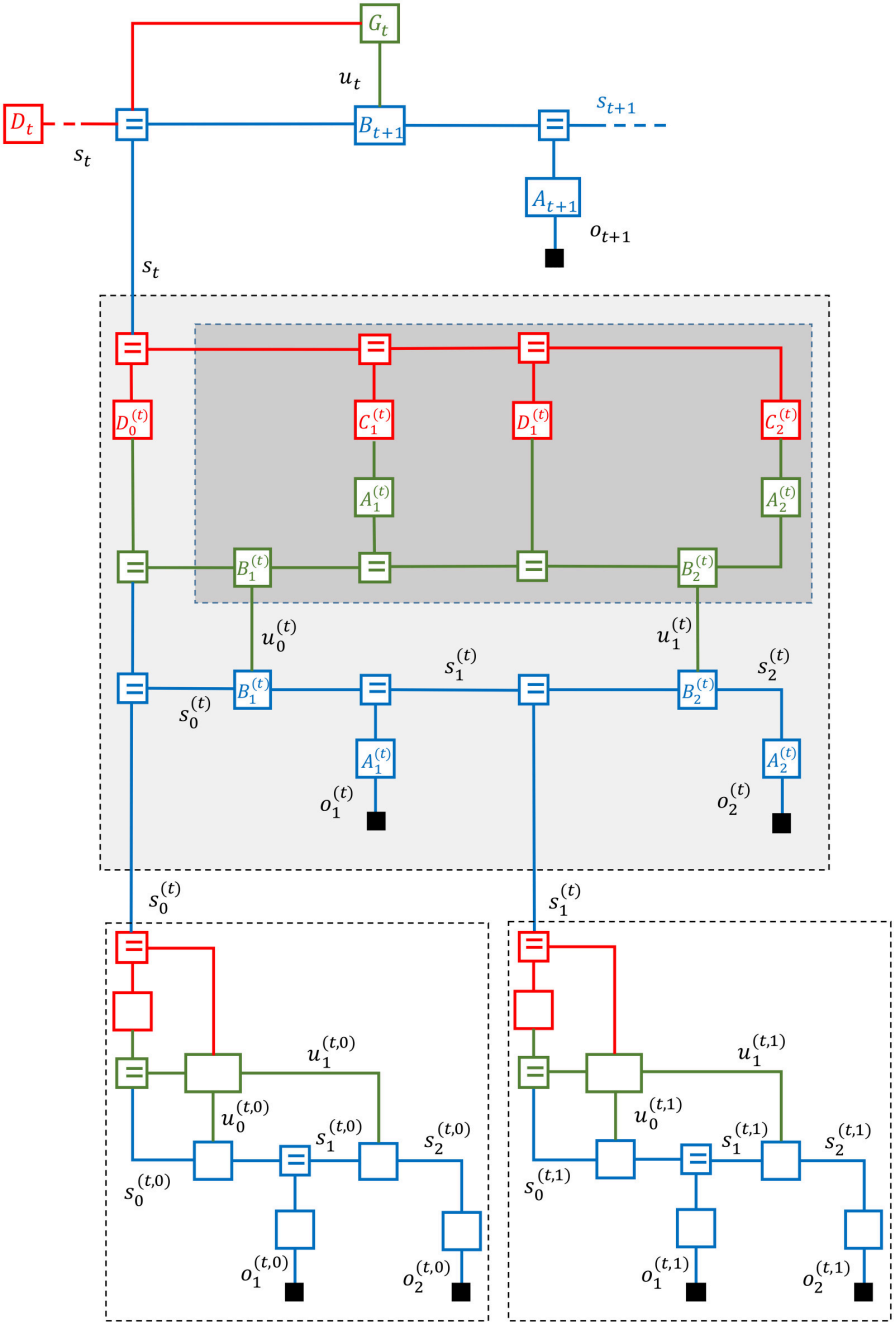
A Forney-style factor graph of the deep temporal active inference model as discussed in Friston et al. (2017c).

**Figure 8:**
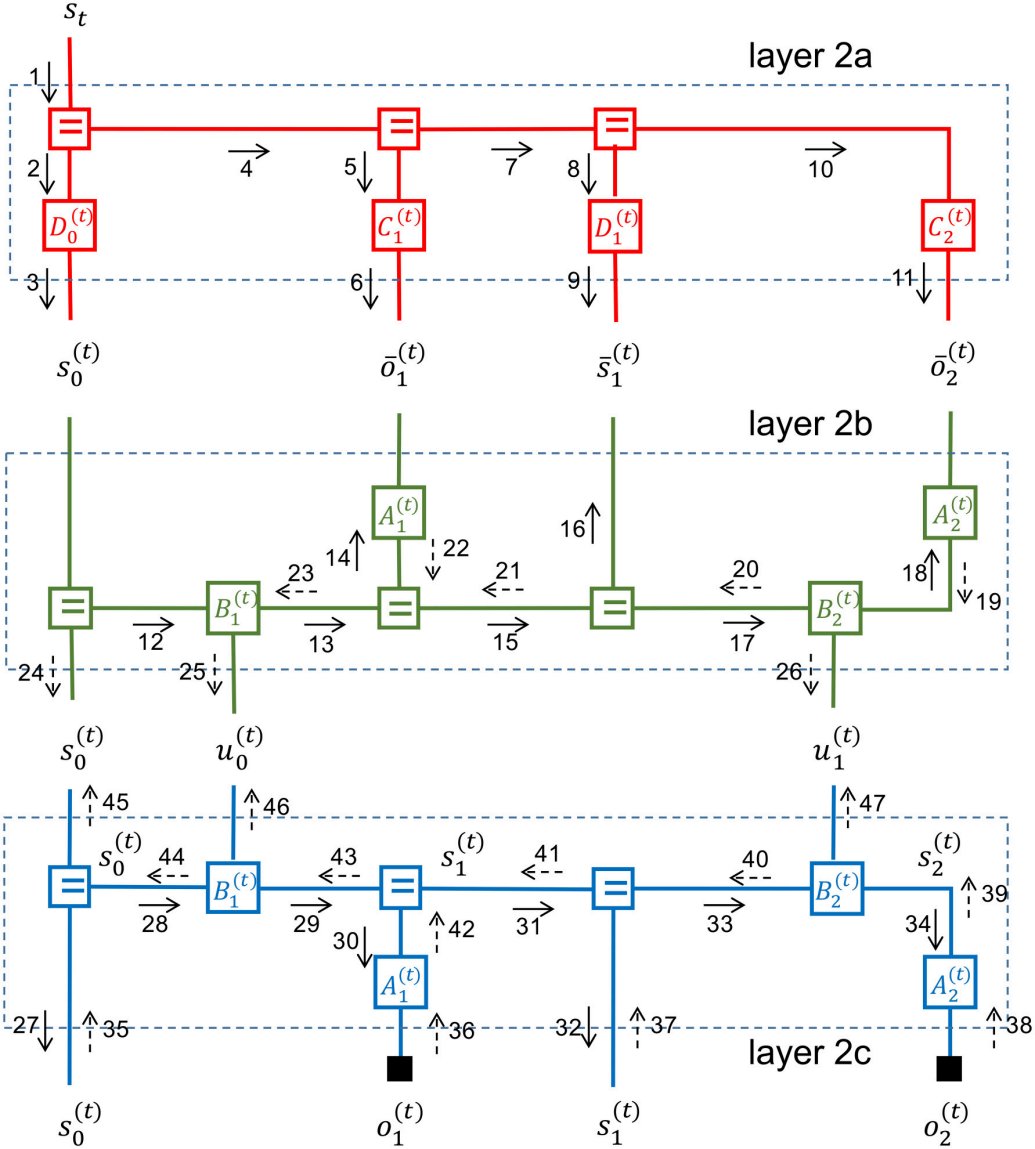
A message passing schedule on the FFG graph for a deep temporal active inference model as discussed in Friston et al. (2017c).

Figure 7 displays a three-layer active inference process with an expected free energy policy model. The policy model for the second layer is enclosed by a dark-shaded rectangle. In Figure 8, the second layer is partitioned into three simple (non-loopy) subgraphs. The top subgraph (layer 2a in red) represents the priors for desired states and observations. The middle subgraph (in green) is a copy of the (blue) state space model that is used by the policy model to simulate the future of the state-space model. The control signals for the bottom subgraph (layer 2c) are inferred through free energy minimization in layers 2a and 2b. This leads to controls that minimize expected free energy. The “generative” policy model is formally specified by

(20) $$\prod_{k=1}^{2}\overset{\text{observation and state priors}}{\overset{︷}{\underset{C_{k}^{\left(t\right)}}{\underset{︸}{p({\bar{o}}_{k}^{\left(t\right)}|s_{t})}}\underset{D_{k-1}^{\left(t\right)}}{\underset{︸}{p({\bar{s}}_{k-1}^{\left(t\right)}|s_{t})}}}}\overset{\text{dynamical system}}{\overset{︷}{\underset{A_{k}^{\left(t\right)}}{\underset{︸}{p({\bar{o}}_{k}^{\left(t\right)}|{\bar{s}}_{k}^{\left(t\right)})}}\underset{B_{k}^{\left(t\right)}}{\underset{︸}{p({\bar{s}}_{k}^{\left(t\right)}|{\bar{s}}_{k-1}^{\left(t\right)},u_{k-1}^{\left(t\right)})}}}}$$

Note that this model does not specify expected free energy directly. Rather, this is a generative model for controls (policies), where a forward inference pass by free energy minimization leads to an expected free energy prior for the policy (see section 5.2). This generative policy model needs to have access to a copy of the dynamic system model in order to simulate the future. In the formal generative model specification, we use the bar-over-variable-name notation for states and observations in the policy model to distinguish these variables from their “mirror” variables in the regular dynamic system.

Equations (18, 20) together specify a generative model for the middle layer of the deep temporal active inference model. The generative model for the full DTAI system is simply the product of the models for all layers.

### 5.2. Inference in the deep temporal active inference model

Figure 8 depicts a partitioning of the middle layer of a deep temporal active inference model into three non-loopy subgraphs. As discussed, in non-loopy graphs, proper Bayesian marginals for hidden variables can be obtained by multiplying the forward and backward messages for each edge. We now discuss a possible inference schedule for the middle layer.

First, the state of the superior layer is used in layer 2a to infer priors for the states and observations by messages 1–11. These priors specify which future state trajectories and observation patterns are desired or rewarding and as such replace the need for external reward functions.

The initial state message 3 is now used by layer 2b to run the dynamical system forward in messages 12–18. In order to compute message 13 (the state transition), the node $B_{1}^{\left(t\right)}$ needs both message 12 and an incoming control message 46 from $u_{0}^{\left(t\right)}$. The control message is initially set to an uninformative message that weighs each control option equally. Alternative priors on admissible controls are also possible. This forward pass leads to predictions for future states and observations.

Next, the outgoing messages from layer 2a are processed by layer 2b as if they were observations for layer 2b. Layer 2b processes these priors by a backward pass (messages 19–23) that leads to updated marginals for the hidden states in layer 2b.

Having inferred the “desired” future hidden states for the dynamic system, layer 2b will now infer appropriate control signals in messages 25–26. These control signals aim to steer the state-transitions in layer 2c to rewarding state trajectories as encoded by the priors in layer 2a.

Messages 24–26 are now processed by layer 2c in a forward pass by messages 27–34 to infer predictions for both in-layer and lower-layer observations. Actual observational evidence is passed into layer 2c by messages 35–38 and further processed in layer 2c by a corrective backward pass through messages 39–44. The backward and forward messages in layer 2c combine to update the marginals of the hidden states.

After the states in layer 2c have been corrected by observational evidence, layer 2c passes the updated information upwards to the initial state and control edges by messages 45–47.

In order to not clutter the figure even more, we have refrained from drawing the messages that push the corrections back through layers 2b, 2a and then upwards to higher layers.

Messages 25 and 26 relate to control signals that minimize the expected free energy in the dynamic system. Messages 46 and 47 encode corrections for these control signals after having observed the evidence. The forward and backward message pairs (25, 46) and (26, 47) multiply to correct the marginals for the controls signals (and similarly 24 and 45 combine to update the marginal for the initial state).

With updated marginals for the initial state and control signals, we can run layer 2b forward again to get corrected predictions for the states ${\overset{-}{s}}_{k}^{\left(t\right)}$ and ${ō}_{k}^{\left(t\right)}$.

Next, layer 2a processes these updated predictions in two ways. The corrections may be partially absorbed by updating the priors for $C_{k}^{\left(t\right)}$ and $D_{k}^{\left(t\right)}$. This is a *learning* step. The remaining free energy is passed on to the superior layer by a backward message for state *s*_*t*_.

Learning of priors serves a similar purpose to learning of the reward function in inverse reinforcement learning algorithms (Ng and Russell, 2000). Crucially, active inference needs no special recipes for learning rewards nor for selecting useful policies. All relevant tasks are accomplished by minimizing free energy in a generative probabilistic model.

In summary, deep temporal active inference can be modeled as a multi-scale hierarchical dynamic system with a particular policy model. Each layer can be partitioned into three non-loopy sub-layers. The FFG formalism provides both an insightful representation and computational mechanisms to execute active inference processes by message passing inside and between the (sub-)layers.

## 6. Discussion

It is interesting to appreciate the symmetry in an active inference layer. Consider the middle layer in Figure 7 again. The blue subgraph is the unfolded state-space model terminated by actual observations $o_{1}^{\left(t\right)}$ and $o_{2}^{\left(t\right)}$. The green subgraph is a copy of the same state-space model, but now terminated by priors *C*^(*t*)^ and *D*^(*t*)^. The backward message stream transfers evidence from both in-layer and lower-layer observations into the priors *C*^(*t*)^ and *D*^(*t*)^. Left-over free energy gets pushed up to higher layers.

If the higher levels would operate at the same time scale as the lower layers, then the backward messages would quickly become uninformative and processing in the high-order layers would not be effective in absorbing surprise. Instead, high-order layers process accumulated surprise over multiple time steps of lower layers. At these larger time scales, the incoming backward messages are informative again and processing of these messages leads to more surprise minimization (relative to processing at the same time scale as the lower layers). Thus, effective surprise minimization of highly structured signals leads naturally to multi-scale hierarchical models.

Updating the hidden states in the network proceeds by forward prediction steps that push down expected (or predicted) free energy and upward correction steps that push up unexplained free energy. The Forney-style factor graph framework breaks down the complete algorithm into small (automatable) local-in-time-and-place message passing steps. Note that the update rules for forward and backward messages are based on the same general (sum-product and variational) update rules. Therefore, the interpretation of surprise minimization as a prediction-correction process is interesting but not relevant to the network itself. Message passing serves only to minimize surprise.

In this paper, we have barely touched upon the learning issues. Learning of purposeful behavior rests upon updating priors for the parameters (*A*, *B*, etc.) of the generative model. In a Bayesian context, parameter updating is conceptually no different from state updating in a dynamical system. As is evident from the Kalman update equations (in particular the sum-product update rule of the equality node), the amount of adaptation of latent variables in dynamic systems depends in subtle ways on the ratio between the precision of the prior-based state prediction and the precision of corrective evidence (likelihood).

These precision variables are (like all variables) represented by edges in FFGs and beliefs over precisions change dynamically through message passing over these edges. In real neural circuits, multiple parallel operating active inference columns may directly affect the dynamic beliefs over precisions in other columns through message passing over lateral connections (Kanai et al., 2015). In an FFG graph, these complex circuits will look like matrices with both horizontal and vertical connections. In order to advance the scientific study of these complex neural structures, it will be necessary to simulate the behavior of these networks in computer simulations. Black-box variational inference toolboxes may not provide any insights in the underlying neuronal surprise minimization mechanisms, while at the same time these networks may be too complex to allow manual derivation of neuronal message passing signals.

In this paper, we have pushed the Forney-style factor graph framework as an alternative candidate formalism to study the behavior of complex neural circuits. FFGs provide an insightful visual representation of factorized probabilistic models. Simple closing-the-box rules lead both to higher visual abstraction levels by creating composite nodes and to message passing-based surprise minimization. Surprise minimization in FFGs is, in principle, automatable in freely definable graphs. Practically, the development of a quality FFG simulation toolbox is not an easy task. A toolbox for simulating inference processes in a wide range of dynamic FFG models is currently under development in our team at TU Eindhoven. We hope to release simulation results of the presented graphs and a first public version of this toolbox somewhere in 2018.

## 7. Conclusions

We have presented a graphical process theory for studying message passing-based surprise minimization in neural circuits. Forney-style factor graphs enjoy already a solid reputation in the coding branches of the information theory community. We think that these graphical models are also eminently suited to support the study of active inference processing in complex neural circuits. To argue our case, we have described a graph for a deep temporal active inference model. The concept of closing-the-box and composite nodes makes it very clear how deep temporal active inference is a special case of a multi-scale hierarchical dynamic system. In particular, the FFG graph shows nicely how *expected* free energy minimization results from a forward inference pass through a generative policy model. We are quite aware that the current paper leaves many open questions, but we hope that this paper generates an interest in the neuroscience community to take a deeper look at factor graphs as describing tools for complex generative neural models.

## Author contributions

All authors listed, have made substantial, direct and intellectual contribution to the work, and approved it for publication.

### Conflict of interest statement

The authors declare that the research was conducted in the absence of any commercial or financial relationships that could be construed as a potential conflict of interest.

## Figures and Tables

**Figure A1:**
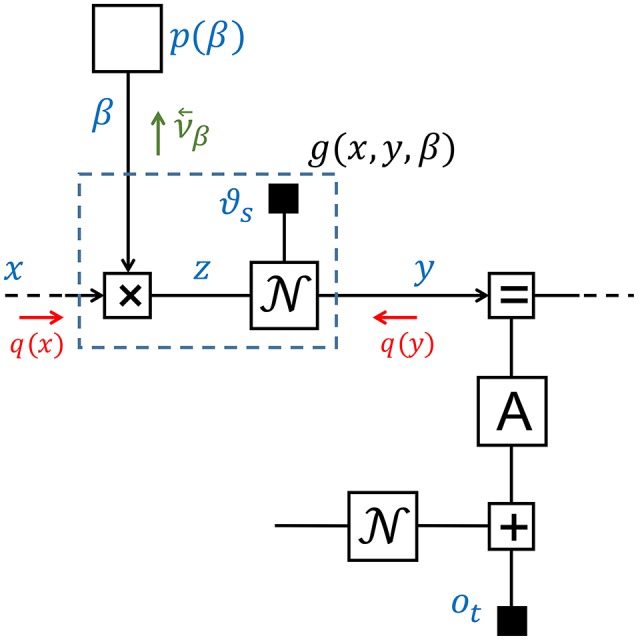
FFG for LGDS with controller *p*(*β*). This drawing is based on Figure 4C with emphasis on the relevant messages for calculating the backward variational message for *β*.

## Footnotes

^1^Technically, a proper model definition would include conditioning on the model specification M, i.o.w. p(z,o|M) is more accurate, but we will ignore the conditioning on M, which is assumed to be present in every expression.

^2^In order to facilitate cross-reading, we follow the notational choices of Friston et al. (2017b) where applicable.

## Appendix A

In Figure A1, we have redrawn Figure 4C with emphasis on the relevant nodes and messages. We will derive a Gaussian variational message ${\overset{⃖}{\nu }}_{\beta }$ that passes information from observations backwards through the state-space model toward the controller node *p*(*β*). We first make a composite node *g*(*x, y*, *β*) that encompasses the multiplier and additive state noise with given variance ϑ_*s*_. Closing the box yields

$$\begin{array}{l} g(x,y,\beta )=\int \delta (z-\beta x)N(y|z,\vartheta_{s})\text{d}z\\ \;=N(y|\beta x,\vartheta_{s}). \end{array}$$

Assume incoming messages $q\left(x\right)=N\left(x|m_{x},\vartheta_{x}\right)$ and $q\left(y\right)=N\left(y|m_{y},\vartheta_{y}\right)$ toward the composite node *g*. The backward variational message for *β* follows from Equation (12):

$$\begin{array}{l} \text{log }{\overset{\leftarrow }{\nu }}_{\beta }\propto E_{q_{x},q_{y}}\left[\text{log }g(x,y,\beta )\right]\\ \;=E_{q_{x},q_{y}}\left[\text{log }N(y|\beta x,\vartheta_{s})\right]\\ \;\propto E_{q_{x},q_{y}}\left[-\frac{{\left(y-\beta x\right)}^{2}}{2\vartheta_{s}}\right]\\ \;\propto -\frac{1}{2\vartheta_{s}}\left(E_{q_{y}}\left[y^{2}\right]-2\beta E_{q_{x}}\left[x\right]E_{q_{y}}\left[y\right]+\beta^{2}E_{q_{x}}\left[x^{2}\right]\right)\\ \;=-\frac{1}{2\vartheta_{s}}\left(\left(m_{y}^{2}+\vartheta_{y}\right)-2\beta m_{x}m_{y}+\beta^{2}\left(m_{x}^{2}+\vartheta_{x}\right)\right)\\ \;\propto -\frac{m_{x}^{2}+\vartheta_{x}}{2\vartheta_{s}}{\left(\beta -\frac{m_{x}m_{y}}{m_{x}^{2}+\vartheta_{x}}\right)}^{2}. \end{array}$$

Hence it follows that

$${\overset{\leftarrow }{\nu }}_{\beta }=N\left(\beta |\frac{m_{x}m_{y}}{m_{x}^{2}+\vartheta_{x}},\frac{\vartheta_{s}}{m_{x}^{2}+\vartheta_{x}}\right).$$
